# [μ-2,2′-(1,4-Phenyl­ene)diacetato-κ^2^ *O* ^1^:*O* ^4^]bis­[aqua­(2,2′-bipyridine-κ^2^ *N*,*N*′)chloridocopper(II)] dihydrate

**DOI:** 10.1107/S1600536812029686

**Published:** 2012-07-25

**Authors:** Jin-He Zhao, Yan-Xia Lin, Wei Wu, Zhong Zhang

**Affiliations:** a Department of Chemical and Life Science, Baise University, Baise 533000, People’s Republic of China; b Hybio Pharmaceutical Co. Ltd, Shenzhen 518057, People’s Republic of China; c Department of Chemistry and Chemical Engineering, Guangxi University for Nationalities, Nanning 530006, People’s Republic of China

## Abstract

In the centrosymmetric title compound, [Cu_2_(C_10_H_8_O_4_)Cl_2_(C_10_H_8_N_2_)_2_(H_2_O)_2_]·2H_2_O, the Cu^II^ atom is five-coordinated in a distorted square-pyramidal geometry by two N atoms from a chelating 2,2′-bipyridine ligand, one O atom from a 1,4-phenyl­enediacetate ligand, one Cl atom and one water mol­ecule. The 1,4-phenyl­enediacetate ligand, lying on an inversion center, bridges two Cu^II^ atoms. In the crystal, O—H⋯O and O—H⋯Cl hydrogen bonds and π–π inter­actions between the pyridine rings [centroid–centroid distance = 3.740 (5) Å] link the complex mol­ecules and uncoordinated water mol­ecules into a three-dimensional network.

## Related literature

For related structures, see: Hu *et al.* (2009 ▶); Wu *et al.* (2010 ▶, 2011 ▶).
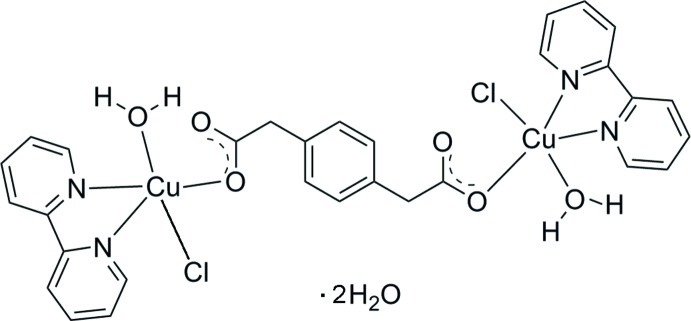


## Experimental

### 

#### Crystal data

[Cu_2_(C_10_H_8_O_4_)Cl_2_(C_10_H_8_N_2_)_2_(H_2_O)_2_]·2H_2_O
*M* *_r_* = 774.58
Monoclinic, 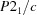

*a* = 11.713 (4) Å
*b* = 6.954 (2) Å
*c* = 19.585 (7) Å
β = 100.507 (5)°
*V* = 1568.6 (9) Å^3^
*Z* = 2
Mo *K*α radiation
μ = 1.58 mm^−1^
*T* = 296 K
0.41 × 0.39 × 0.37 mm

#### Data collection

Bruker SMART 1000 CCD diffractometer
Absorption correction: multi-scan (*SADABS*; Sheldrick, 1996 ▶) *T*_min_ = 0.563, *T*_max_ = 0.592
7958 measured reflections
3011 independent reflections
2394 reflections with *I* > 2σ(*I*)
*R*_int_ = 0.039

#### Refinement

*R*[*F* ^2^ > 2σ(*F* ^2^)] = 0.083
*wR*(*F* ^2^) = 0.177
*S* = 1.06
3011 reflections
209 parameters
H-atom parameters constrained
Δρ_max_ = 0.92 e Å^−3^
Δρ_min_ = −0.79 e Å^−3^

### 

Data collection: *SMART* (Bruker, 2007 ▶); cell refinement: *SAINT* (Bruker, 2007 ▶); data reduction: *SAINT*; program(s) used to solve structure: *SHELXS97* (Sheldrick, 2008 ▶); program(s) used to refine structure: *SHELXL97* (Sheldrick, 2008 ▶); molecular graphics: *DIAMOND* (Brandenburg, 1999 ▶); software used to prepare material for publication: *SHELXTL* (Sheldrick, 2008 ▶).

## Supplementary Material

Crystal structure: contains datablock(s) I, global. DOI: 10.1107/S1600536812029686/hy2565sup1.cif

Structure factors: contains datablock(s) I. DOI: 10.1107/S1600536812029686/hy2565Isup2.hkl

Supplementary material file. DOI: 10.1107/S1600536812029686/hy2565Isup3.cdx

Additional supplementary materials:  crystallographic information; 3D view; checkCIF report

## Figures and Tables

**Table 1:** Hydrogen-bond geometry (Å, °)

*D*—H⋯*A*	*D*—H	H⋯*A*	*D*⋯*A*	*D*—H⋯*A*
O3—H3*A*⋯O2^i^	0.85	2.00	2.822 (8)	164
O3—H3*B*⋯O4^ii^	0.85	1.94	2.769 (8)	164
O4—H4*A*⋯O1	0.85	2.06	2.900 (8)	169
O4—H4*B*⋯Cl1^ii^	0.85	2.44	3.276 (7)	169

Symmetry codes: (i) 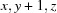; (ii) 
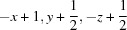
.

**Fig. 1.**
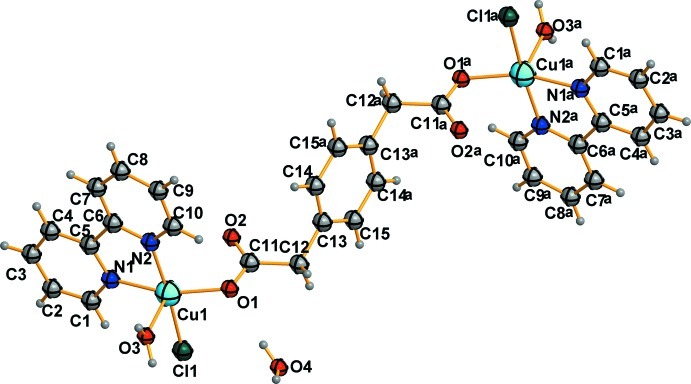
The molecular structure of the title compound, showing 50% probability ellipsoids. [Symmetry code: (a) 1-x, -y, 1-z.]

**Fig. 2.**
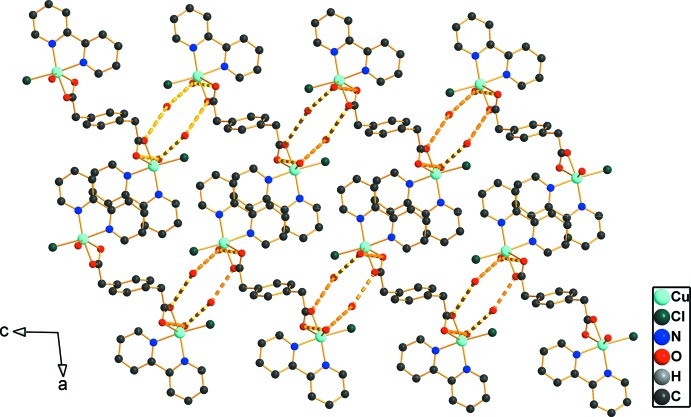
Crystal packing of the title compound, viewed along the b axis. Hydrogen bonds are shown as dashed lines.

## supplementary crystallographic information

### Comment

2,2'-Bipyridine (2,2'-bpy) is one of the first-line popular drugs used to treat invasive infections. 1,4-Phenylenediacetic acid (H_2_PDA) has two flexible acetate groups, resulting in *trans*- or *cis*-conformation. Both of them are interesting candidates to coordinate to metal ions through nitrogen atoms or oxygen atoms for constructing a diversity of coordination architectures (Hu *et al.*, 2009; Wu *et al.*, 2010, 2011).

In the title complex (Fig. 1), the Cu^II^ atom is five-coordinated by two N atoms (N1, N2) from a 2,2-bpy ligand, one O atom (O1) from a centrosymmetric PDA ligand, one O atom (O3) from a water molecule and one chloride ion. The asymmetric unit also contains one uncoordinated water molecule. O—H···O and O—H···Cl hydrogen bonds (Table 1) and π–π interactions between the pyridine rings [centroid–centroid distance = 3.740 (5) Å] result in the formation of a supramolecular structure (Fig. 2).

### Experimental

1,4-Phenylenediacetic acid (0.097 g, 0.5 mmol) and 2,2-bipyridine (0.078 g, 0.5 mmol) were dissolved in a mixture of 15 ml N,N-dimethylformamide and 10 ml water and an aqueous solution of sodium hydroxide was added dropwise with stirring to adjust the pH value being 6. Then 5 ml aqueous solution of CuCl_2_.2H_2_O (0.097 g, 0.05 mmol) was added. The mixture was kept stirring at 350 K for 4 h and then filtered. The filtrate was kept at room temperature and a few days later X-ray quality blue block-shaped crystals were obtained. Analysis, calculated for C_30_H_32_Cl_2_Cu_2_N_4_O_8_: C 46.52, H 4.16, N 7.23%; found: C 46.49, H 4.17, N 7.21%.

### Refinement

H atoms bonded to O atoms were located in a difference Fourier map and refined as riding atoms, with O—H = 0.85 Å and with *U*_iso_(H) = 1.2*U*_eq_(O). Other H atoms were positioned geometrically and refined using a riding model, with C—H = 0.93 (aromatic) and 0.97 (CH_2_) Å and with *U*_iso_(H) = 1.2*U*_eq_(C).

### Crystal data

**Table:**

[Cu_2_(C_10_H_8_O_4_)Cl_2_(C_10_H_8_N_2_)_2_(H_2_O)_2_]·2H_2_O	*F*(000) = 792
*M**_r_* = 774.58	*D*_x_ = 1.640 Mg m^−^^3^
Monoclinic, *P*2_1_/*c*	Mo *K*α radiation, λ = 0.71073 Å
Hall symbol: -P 2ybc	Cell parameters from 3637 reflections
*a* = 11.713 (4) Å	θ = 2.5–28.2°
*b* = 6.954 (2) Å	µ = 1.58 mm^−^^1^
*c* = 19.585 (7) Å	*T* = 296 K
β = 100.507 (5)°	Block, blue
*V* = 1568.6 (9) Å^3^	0.41 × 0.39 × 0.37 mm
*Z* = 2	

### Data collection

**Table:**

Bruker SMART 1000 CCD diffractometer	3011 independent reflections
Radiation source: fine-focus sealed tube	2394 reflections with *I* > 2σ(*I*)
Graphite monochromator	*R*_int_ = 0.039
φ and ω scans	θ_max_ = 26.0°, θ_min_ = 1.8°
Absorption correction: multi-scan (*SADABS*; Sheldrick, 1996)	*h* = −14→14
*T*_min_ = 0.563, *T*_max_ = 0.592	*k* = −8→8
7958 measured reflections	*l* = −23→18

### Refinement

**Table:**

Refinement on *F*^2^	Secondary atom site location: difference Fourier map
Least-squares matrix: full	Hydrogen site location: inferred from neighbouring sites
*R*[*F*^2^ > 2σ(*F*^2^)] = 0.083	H-atom parameters constrained
*wR*(*F*^2^) = 0.177	*w* = 1/[σ^2^(*F*_o_^2^) + (0.0067*P*)^2^ + 24.0162*P*] where *P* = (*F*_o_^2^ + 2*F*_c_^2^)/3
*S* = 1.06	(Δ/σ)_max_ < 0.001
3011 reflections	Δρ_max_ = 0.92 e Å^−^^3^
209 parameters	Δρ_min_ = −0.79 e Å^−^^3^
0 restraints	Extinction correction: *SHELXL97* (Sheldrick, 2008), Fc^*^=kFc[1+0.001xFc^2^λ^3^/sin(2θ)]^-1/4^
Primary atom site location: structure-invariant direct methods	Extinction coefficient: 0.0220 (15)

### Special details

**Table:**

Geometry. All e.s.d.'s (except the e.s.d. in the dihedral angle between two l.s. planes) are estimated using the full covariance matrix. The cell e.s.d.'s are taken into account individually in the estimation of e.s.d.'s in distances, angles and torsion angles; correlations between e.s.d.'s in cell parameters are only used when they are defined by crystal symmetry. An approximate (isotropic) treatment of cell e.s.d.'s is used for estimating e.s.d.'s involving l.s. planes.
Refinement. Refinement of *F*^2^ against ALL reflections. The weighted *R*-factor *wR* and goodness of fit *S* are based on *F*^2^, conventional *R*-factors *R* are based on *F*, with *F* set to zero for negative *F*^2^. The threshold expression of *F*^2^ > σ(*F*^2^) is used only for calculating *R*-factors(gt) *etc*. and is not relevant to the choice of reflections for refinement. *R*-factors based on *F*^2^ are statistically about twice as large as those based on *F*, and *R*- factors based on ALL data will be even larger.

### Fractional atomic coordinates and isotropic or equivalent isotropic displacement parameters (Å^2^)

**Table:**

	*x*	*y*	*z*	*U*_iso_*/*U*_eq_	
Cu1	0.77380 (7)	0.64270 (13)	0.35400 (4)	0.0243 (3)	
Cl1	0.71866 (18)	0.5594 (4)	0.24104 (10)	0.0431 (6)	
N1	0.9450 (5)	0.6443 (10)	0.3524 (3)	0.0313 (14)	
N2	0.8374 (5)	0.7190 (9)	0.4531 (3)	0.0281 (14)	
O1	0.6232 (4)	0.5601 (8)	0.3742 (3)	0.0295 (12)	
O2	0.7159 (4)	0.2915 (8)	0.4109 (3)	0.0345 (13)	
O3	0.7324 (6)	0.9562 (8)	0.3321 (3)	0.0473 (16)	
H3A	0.7360	1.0454	0.3620	0.057*	
H3B	0.6885	0.9940	0.2951	0.057*	
O4	0.4144 (6)	0.6431 (10)	0.2741 (3)	0.0547 (18)	
H4A	0.4750	0.6346	0.3053	0.066*	
H4B	0.3871	0.7562	0.2748	0.066*	
C1	0.9972 (8)	0.6049 (13)	0.2969 (4)	0.041 (2)	
H1	0.9499	0.5767	0.2546	0.049*	
C2	1.1147 (8)	0.6046 (15)	0.2998 (5)	0.048 (2)	
H2	1.1470	0.5736	0.2612	0.057*	
C3	1.1824 (7)	0.6517 (14)	0.3616 (4)	0.041 (2)	
H3	1.2625	0.6590	0.3649	0.050*	
C4	1.1348 (7)	0.6885 (12)	0.4192 (4)	0.0337 (18)	
H4	1.1818	0.7147	0.4618	0.040*	
C5	1.0153 (6)	0.6858 (9)	0.4124 (4)	0.0235 (15)	
C6	0.9531 (6)	0.7264 (10)	0.4706 (4)	0.0224 (15)	
C7	1.0085 (7)	0.7747 (11)	0.5371 (4)	0.0295 (17)	
H7	1.0891	0.7797	0.5483	0.035*	
C8	0.9419 (8)	0.8147 (11)	0.5861 (4)	0.0360 (19)	
H8	0.9771	0.8440	0.6313	0.043*	
C9	0.8234 (7)	0.8113 (13)	0.5681 (4)	0.039 (2)	
H9	0.7774	0.8423	0.6005	0.047*	
C10	0.7729 (6)	0.7610 (12)	0.5006 (4)	0.0318 (17)	
H10	0.6924	0.7565	0.4884	0.038*	
C11	0.6254 (6)	0.3871 (10)	0.3943 (3)	0.0240 (15)	
C12	0.5077 (6)	0.2956 (12)	0.3957 (4)	0.0316 (18)	
H12A	0.4540	0.3965	0.4031	0.038*	
H12B	0.4790	0.2399	0.3504	0.038*	
C13	0.5059 (6)	0.1417 (11)	0.4501 (4)	0.0245 (15)	
C14	0.5399 (7)	0.1820 (11)	0.5198 (4)	0.0330 (18)	
H14	0.5661	0.3047	0.5336	0.040*	
C15	0.4647 (6)	−0.0410 (11)	0.4305 (4)	0.0282 (16)	
H15	0.4399	−0.0693	0.3838	0.034*	

### Atomic displacement parameters (Å^2^)

**Table:**

	*U*^11^	*U*^22^	*U*^33^	*U*^12^	*U*^13^	*U*^23^
Cu1	0.0217 (5)	0.0281 (5)	0.0225 (5)	−0.0032 (4)	0.0024 (3)	−0.0004 (4)
Cl1	0.0393 (11)	0.0609 (14)	0.0257 (10)	−0.0081 (10)	−0.0029 (8)	−0.0083 (9)
N1	0.034 (3)	0.033 (3)	0.026 (3)	−0.004 (3)	0.003 (3)	0.006 (3)
N2	0.027 (3)	0.030 (3)	0.026 (3)	−0.003 (3)	0.002 (3)	−0.001 (3)
O1	0.021 (3)	0.033 (3)	0.033 (3)	−0.001 (2)	0.002 (2)	0.004 (2)
O2	0.029 (3)	0.034 (3)	0.039 (3)	0.002 (2)	0.002 (2)	0.007 (2)
O3	0.074 (4)	0.028 (3)	0.037 (3)	0.013 (3)	0.002 (3)	0.002 (3)
O4	0.053 (4)	0.044 (4)	0.058 (4)	0.008 (3)	−0.014 (3)	−0.003 (3)
C1	0.045 (5)	0.051 (5)	0.025 (4)	−0.004 (4)	0.004 (4)	0.001 (4)
C2	0.038 (5)	0.069 (7)	0.041 (5)	−0.004 (5)	0.018 (4)	0.005 (5)
C3	0.025 (4)	0.061 (6)	0.035 (4)	−0.004 (4)	−0.002 (3)	0.007 (4)
C4	0.027 (4)	0.040 (5)	0.033 (4)	−0.001 (3)	0.003 (3)	−0.003 (4)
C5	0.022 (3)	0.016 (3)	0.032 (4)	−0.005 (3)	0.005 (3)	0.005 (3)
C6	0.020 (3)	0.020 (3)	0.026 (4)	0.003 (3)	0.000 (3)	0.002 (3)
C7	0.029 (4)	0.028 (4)	0.028 (4)	−0.007 (3)	−0.004 (3)	0.000 (3)
C8	0.053 (5)	0.031 (4)	0.023 (4)	0.004 (4)	0.003 (3)	−0.007 (3)
C9	0.039 (5)	0.047 (5)	0.035 (4)	0.001 (4)	0.018 (4)	−0.010 (4)
C10	0.023 (4)	0.042 (5)	0.031 (4)	0.002 (3)	0.007 (3)	0.002 (4)
C11	0.036 (4)	0.021 (4)	0.014 (3)	−0.005 (3)	0.000 (3)	0.001 (3)
C12	0.023 (4)	0.041 (4)	0.026 (4)	−0.012 (3)	−0.009 (3)	0.003 (3)
C13	0.018 (3)	0.034 (4)	0.022 (3)	−0.002 (3)	0.007 (3)	0.005 (3)
C14	0.043 (5)	0.024 (4)	0.032 (4)	−0.002 (3)	0.006 (3)	−0.006 (3)
C15	0.029 (4)	0.033 (4)	0.021 (3)	−0.010 (3)	0.001 (3)	−0.001 (3)

### Geometric parameters (Å, º)

**Table:**

Cu1—O1	1.963 (5)	C4—C5	1.383 (10)
Cu1—N1	2.011 (6)	C4—H4	0.9300
Cu1—N2	2.020 (6)	C5—C6	1.487 (10)
Cu1—O3	2.257 (6)	C6—C7	1.387 (9)
Cu1—Cl1	2.264 (2)	C7—C8	1.369 (11)
N1—C5	1.338 (9)	C7—H7	0.9300
N1—C1	1.368 (10)	C8—C9	1.368 (12)
N2—C10	1.333 (10)	C8—H8	0.9300
N2—C6	1.338 (9)	C9—C10	1.391 (11)
O1—C11	1.265 (8)	C9—H9	0.9300
O2—C11	1.243 (9)	C10—H10	0.9300
O3—H3A	0.8500	C11—C12	1.523 (10)
O3—H3B	0.8500	C12—C13	1.513 (10)
O4—H4A	0.8500	C12—H12A	0.9700
O4—H4B	0.8500	C12—H12B	0.9700
C1—C2	1.367 (12)	C13—C14	1.379 (10)
C1—H1	0.9300	C13—C15	1.388 (10)
C2—C3	1.361 (12)	C14—C15^i^	1.389 (11)
C2—H2	0.9300	C14—H14	0.9300
C3—C4	1.369 (11)	C15—C14^i^	1.389 (11)
C3—H3	0.9300	C15—H15	0.9300
			
O1—Cu1—N1	160.0 (2)	C4—C5—C6	123.6 (7)
O1—Cu1—N2	94.1 (2)	N2—C6—C7	121.9 (7)
N1—Cu1—N2	79.6 (3)	N2—C6—C5	114.3 (6)
O1—Cu1—O3	98.7 (2)	C7—C6—C5	123.8 (6)
N1—Cu1—O3	99.9 (3)	C8—C7—C6	118.6 (7)
N2—Cu1—O3	87.6 (2)	C8—C7—H7	120.7
O1—Cu1—Cl1	90.87 (16)	C6—C7—H7	120.7
N1—Cu1—Cl1	95.37 (19)	C9—C8—C7	119.8 (7)
N2—Cu1—Cl1	174.95 (19)	C9—C8—H8	120.1
O3—Cu1—Cl1	92.75 (17)	C7—C8—H8	120.1
C5—N1—C1	116.6 (7)	C8—C9—C10	119.0 (7)
C5—N1—Cu1	116.3 (5)	C8—C9—H9	120.5
C1—N1—Cu1	127.1 (5)	C10—C9—H9	120.5
C10—N2—C6	119.4 (6)	N2—C10—C9	121.4 (7)
C10—N2—Cu1	124.9 (5)	N2—C10—H10	119.3
C6—N2—Cu1	115.8 (5)	C9—C10—H10	119.3
C11—O1—Cu1	111.9 (5)	O2—C11—O1	123.9 (7)
Cu1—O3—H3A	126.3	O2—C11—C12	120.2 (6)
Cu1—O3—H3B	122.4	O1—C11—C12	115.8 (7)
H3A—O3—H3B	108.0	C13—C12—C11	115.9 (6)
H4A—O4—H4B	108.7	C13—C12—H12A	108.3
C2—C1—N1	124.1 (8)	C11—C12—H12A	108.3
C2—C1—H1	118.0	C13—C12—H12B	108.3
N1—C1—H1	118.0	C11—C12—H12B	108.3
C3—C2—C1	117.1 (8)	H12A—C12—H12B	107.4
C3—C2—H2	121.5	C14—C13—C15	118.9 (7)
C1—C2—H2	121.5	C14—C13—C12	121.0 (7)
C2—C3—C4	121.2 (8)	C15—C13—C12	120.1 (6)
C2—C3—H3	119.4	C13—C14—C15^i^	120.6 (7)
C4—C3—H3	119.4	C13—C14—H14	119.7
C3—C4—C5	118.4 (7)	C15^i^—C14—H14	119.7
C3—C4—H4	120.8	C13—C15—C14^i^	120.6 (7)
C5—C4—H4	120.8	C13—C15—H15	119.7
N1—C5—C4	122.5 (7)	C14^i^—C15—H15	119.7
N1—C5—C6	114.0 (6)		

Symmetry code: (i) −*x*+1, −*y*, −*z*+1.

### Hydrogen-bond geometry (Å, º)

**Table:**

*D*—H···*A*	*D*—H	H···*A*	*D*···*A*	*D*—H···*A*
O3—H3*A*···O2^ii^	0.85	2.00	2.822 (8)	164
O3—H3*B*···O4^iii^	0.85	1.94	2.769 (8)	164
O4—H4*A*···O1	0.85	2.06	2.900 (8)	169
O4—H4*B*···Cl1^iii^	0.85	2.44	3.276 (7)	169

Symmetry codes: (ii) *x*, *y*+1, *z*; (iii) −*x*+1, *y*+1/2, −*z*+1/2.

## References

[bb1] Brandenburg, K. (1999). *DIAMOND* Crystal Impact GbR, Bonn, Germany.

[bb2] Bruker (2007). *SMART* and *SAINT* Bruker AXS Inc., Madison, Wisconsin, USA.

[bb3] Hu, F.-L., Yin, X.-H., Mi, Y., Zhang, J.-L., Zhuang, Y. & Dai, X.-Z. (2009). *Inorg. Chem. Commun.* **12**, 628–631.

[bb4] Sheldrick, G. M. (1996). *SADABS* University of Göttingen, Germany.

[bb5] Sheldrick, G. M. (2008). *Acta Cryst.* A**64**, 112–122. 10.1107/S0108767307043930 18156677

[bb6] Wu, Q.-L., Luo, Z.-R., Zhuang, J.-C. & Yin, X.-H. (2011). *J. Chem. Crystallogr.* **41**, 664–669.

[bb7] Wu, Q.-L., Zhuang, J.-C., Luo, Z.-R., Yin, X.-H. & Zhao, D.-D. (2010). *Synth. React. Inorg. Met. Org. Nano-Met. Chem.* **40**, 790–797.

